# Deep learning-empowered crop breeding: intelligent, efficient and promising

**DOI:** 10.3389/fpls.2023.1260089

**Published:** 2023-10-03

**Authors:** Xiaoding Wang, Haitao Zeng, Limei Lin, Yanze Huang, Hui Lin, Youxiong Que

**Affiliations:** ^1^ Fujian Provincial Key Lab of Network Security & Cryptology, College of Computer and Cyber Security, Fujian Normal University, Fuzhou, China; ^2^ School of Computer Science and Mathematics, Fujian Provincial Key Laboratory of Big Data Mining and Applications, Fujian University of Technology, Fuzhou, China; ^3^ Key Laboratory of Sugarcane Biology and Genetic Breeding, Ministry of Agriculture and Rural Affairs, Fujian Agriculture and Forestry University, Fuzhou, China; ^4^ National Key Laboratory for Tropical Crop Breeding, Institute of Tropical Bioscience and Biotechnology, Chinese Academy of Tropical Agricultural Sciences, Hainan, China

**Keywords:** crop breeding, deep learning, smart breeding, challenge, prospect

## Abstract

Crop breeding is one of the main approaches to increase crop yield and improve crop quality. However, the breeding process faces challenges such as complex data, difficulties in data acquisition, and low prediction accuracy, resulting in low breeding efficiency and long cycle. Deep learning-based crop breeding is a strategy that applies deep learning techniques to improve and optimize the breeding process, leading to accelerated crop improvement, enhanced breeding efficiency, and the development of higher-yielding, more adaptive, and disease-resistant varieties for agricultural production. This perspective briefly discusses the mechanisms, key applications, and impact of deep learning in crop breeding. We also highlight the current challenges associated with this topic and provide insights into its future application prospects.

## Introduction

Crop quality has always been a focal point in human cultivation, and crop breeding, as a primary approach to increasing crop yield and improving crop quality, is one of the oldest agricultural activities, equivalent to human civilization (Shen et al., 2022). Emerging as the times require, crop breeding is the process of artificially selecting and cultivating plant varieties to improve their agronomic traits and economic benefits (Herath et al., 2021). In the early days, farmers preserved and planted the seeds of the best-performing plants to grow crops in the next season, a natural selection process that facilitated the accumulation of favorable traits (Ibe, 2022). Over time, people gradually realized the importance of specific traits for crop yield, quality, disease resistance, and adaptability, and began consciously to select and breed plants with these characteristics. With the development of technology and improvement of living standards, higher demands have been placed on crop yield and quality, necessitating continuous innovation in breeding techniques, methods, and applications to provide strong support (Wallace et al., 2018; Jiang et al., 2020). This has significant impacts and effects on agriculture and the economy, promoting sustainable agricultural development.

During the entire history of crop breeding technology, it has roughly gone through three major stages, and it is now advancing towards the fourth stage. The first stage is conventional breeding (Breeding 1.0), which mainly relies on visual observation of crop phenotypes and subjective selection of crops that meet predetermined requirements. Generally, wild species are gradually domesticated into cultivated varieties with improved qualities through multiple rounds of artificial selection (Khoshbakht and Hammer, 2008; Moose and Mumm, 2008). However, this stage primarily relies on natural variation and the subjective experience of breeders, resulting in slow progress, low efficiency, and high uncertainty. In the late 19th century, with the rapid development of genetics, genetic breeding (Breeding 2.0) emerged as the mainstream, bringing breeding into the realm of science. During this stage, significant success was achieved in crop breeding for crops like wheat, rice, maize, greatly improving yields. Unfortunately, there were still shortcomings such as long breeding cycles, low efficiency in genetic improvement, and high field costs (Zhang et al., 2014; Abdallah et al., 2015). At the end of the 20th century, genetic engineering propelled the development of modern molecular biology, ushering in the era of molecular breeding (Breeding 3.0). The gradual application of technologies such as transgenic techniques, molecular markers, genomic selection, and gene editing provided more efficient, precise, and targeted breeding methods. Nevertheless, high costs and complexity remain limiting factors for the application of molecular breeding (Jing et al., 2021). Breeding scientists sincerely hope that the integration of new generation information technologies such as big data and artificial intelligence with biotechnology will propel crop breeding into the era of Breeding 4.0, which also terms as Intelligent breeding (Wang et al., 2020) and is marked by Deep Learning-Empowered Crop Breeding (Yang et al., 2020; Wang et al., 2023).

**Figure 1:**
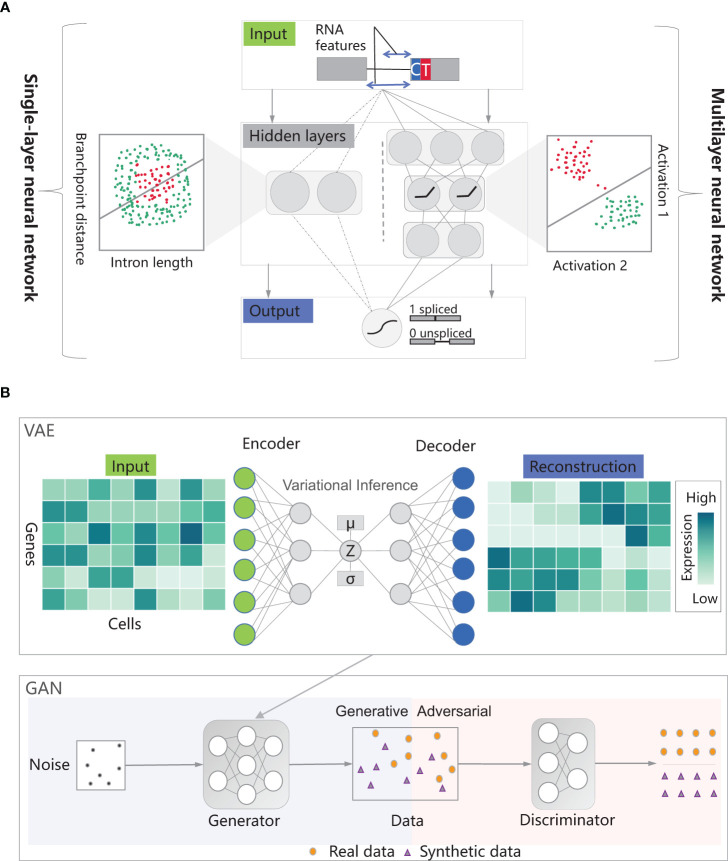
The modes in deep learning. **(A)** The figure shows an example of splice site classification using a single-layer neural network with sigmoid activation. It predicts the probability of output being class 1 based on two RNA features. The goal is to distinguish spliced-out from non-spliced-out introns based on intron length and branchpoint distance. If the length or distance is too short or too long, splicing doesn’t occur. Not surprisingly, it can’t separate the spliced (red) and unspliced (green) data points. In a multilayer neural network, hidden layers in neural networks transform inputs with nonlinear transformations, making classes linearly separable. **(B)** An autoencoder consists of an encoder and a decoder, used to compress input data into a lower-dimensional bottleneck layer for reconstruction. The accuracy of reconstruction is measured using a loss function, enhancing the clarity of the data structure. An autoencoder is endowed with the function of variational inference to form a variational autoencoder. A generative adversarial network include a generator and a discriminator. The generator and discriminator of a generative adversarial network play games with each other, and after the generator is replaced by a variational autoencoder, the generative adversarial network can generate more realistic data. Thanks to the insights from Larsen et al. (2016); Eraslan et al. (2019), and Shete et al. (2020).

With the push of large-scale datasets, powerful computing capabilities, and algorithmic improvements, deep learning has made breakthrough progress in multiple fields (Khan et al., 2019). Deep learning is a machine learning method that revolves around the idea of building multi-layer neural network models to simulate the neural networks of the human brain, enabling the learning and pattern recognition abilities of data, which can be further applied to tasks such as classification, prediction, and generation (LeCun et al., 2015). Depending on whether the training data has label information, deep learning can be divided into two learning modes (Cunningham et al., 2008): (1) Supervised Learning: It relies on labeled training data, where labels correspond to the expected outputs or categories for each input sample. In this mode, explicit supervision signals are provided to the model, enabling it to learn the mapping relationship between inputs and outputs. The algorithms include Convolutional Neural Networks (CNNs), Recurrent Neural Networks (RNNs), and Graph Convolutional Neural Networks (GCNs) (Yan and Wang, 2022). Neural networks are models used to capture nonlinear dependencies. They transform inputs through hidden layers, mapping them to a space where classes can be linearly separated. In the example of splice site classification, a singlelayer neural network employs logistic regression for prediction but fails to accurately differentiate spliced and unspliced data points. Surprisingly, by utilizing neural networks with intermediate layers, more complex nonlinear transformations can be performed, enabling effective discrimination between spliced and unspliced data points (**Figure 1A**). Deep neural networks, on the other hand, are neural network architectures that consist of multiple hidden layers (Miikkulainen et al., 2019). (2) Unsupervised Learning: It utilizes unlabeled training data. In this case, no explicit output or category information is given, and the goal is to discover hidden structures, patterns, or features from the data through the model’s own learning process (Hastie et al., 2009). Unsupervised learning is commonly used for tasks such as clustering, dimensionality reduction, anomaly detection, and generative modeling (Fan et al., 2020). The algorithms include Autoencoders, Generative Adversarial Networks (GANs) and Variational AutoEncoders (VAEs). An autoencoder consists of an encoder and a decoder, used for data compression and reconstruction (Bank et al., 2020). The encoder compresses input data into lower dimensions and stores it in the bottleneck layer, while the decoder attempts to reconstruct the original input from the compressed data in the bottleneck layer. A generative adversarial network consists of a generator and a discriminator, trained together to generate realistic samples and perform discrimination (Creswell et al., 2018). The discriminator is responsible for distinguishing between real and synthetic data, while the generator aims to deceive the discriminator by generating more realistic synthetic samples (**Figure 1B**).

Deep learning-empowered breeding is a method that applies deep learning techniques to improve and optimize the breeding process. It utilizes deep learning models to analyze and process agricultural and genetic data, in order to predict and optimize the agricultural characteristics and genetic traits of crops (Uzal et al., 2018). Deep learning-assisted breeding can enhance breeding efficiency, accelerate the improvement process of crops or animals, and provide higher-yielding, more adaptive, and disease-resistant varieties for agricultural production, through steps such as data collection and preprocessing, model construction and training, as well as genetic parameter optimization and selection (Liu and Wang, 2017). The aim of this perspective is to provide an overview of the latest developments in deep learning in the field of crop breeding, analyze current challenges, and highlight its potential as a promising technology for crop breeding.

## Principles of deep learning-empowered crop breeding

Deep learning solves complex problems by processing large-scale data. Currently, images remain the main data format for phenotypic selection in crop breeding (Araus and Cairns, 2014). The application of deep learning in plant phenotyping image processing is rapidly advancing, especially with the impressive performance of CNN in analyzing phenotype big data (Chang et al., 2016). It possesses powerful feature extraction and modeling capabilities, providing new approaches for overcoming challenges in data analysis. The workflow for crop breeding based on deep learning generally includes six steps: (1) Data collection: Gather agricultural and genetic data relevant to the target crops, including phenotypic traits, environmental factors, genetic markers, and other related information (Crossa et al., 2010). (2) Data preprocessing: Clean and preprocess the collected data, involving data normalization, feature extraction, missing data handling, and data augmentation techniques (SChadt et al., 2001). (3) Model construction: Build a deep learning model suitable for the specific breeding task, selecting appropriate neural network architectures based on the data nature and breeding objectives. (4) Model training: Train the deep learning model using preprocessed data, optimizing model parameters to minimize differences between predicted outputs and observed values. Training typically involves techniques like backpropagation and gradient descent to update model weights (Zhou, 2018). (5) Genetic parameter optimization and selection: Utilize the trained deep learning model to predict and evaluate agricultural characteristics and genetic traits of crops. Optimize genetic parameters and select suitable individuals for further breeding based on the predictions. (6) Iterative improvement: Repeat the training, evaluation, and selection steps, iteratively improving the deep learning model and breeding process (Ni et al., 2019). This establishes an effective breeding plan, enabling the offspring of parental generations to approach the desired phenotypes.

**Figure 2:**
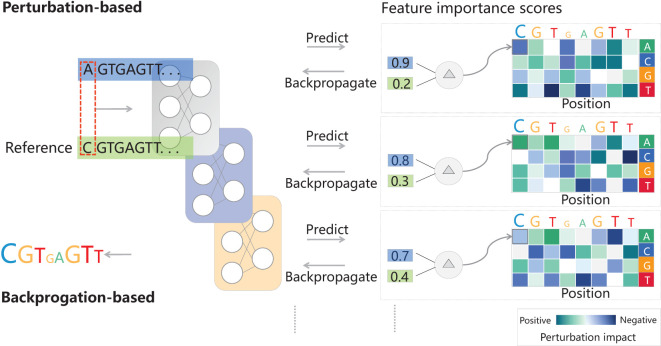
Model interpretation via feature importance scores. It highlights predictive parts of the input. For DNA sequence models, they can be visualized as a sequence logo with letter heights proportional to the scores. Negative scores are shown with upside-down letters. There are two types of importance scores: perturbation-based and backpropagation-based. The methods calculating perturbation-based scores can modify input features, record prediction changes, and create an importance matrix. For DNA sequences, perturbations involve single base substitutions. Therefore, the perturbation matrix can also be visualized as a sequence logo showing average per-base impact. On the other hand, the methods computing backpropagation-based scores normally use gradients or augmented gradients like DeepLIFT (Shrikumar et al., 2017) for input features and model prediction. Thanks to the insights from Simonyan et al. (2013); Shrikumar et al. (2017), and Eraslan et al. (2019).

Deep learning-empowered breeding is built upon important identified genes, integrating multiomics, next-generation biotechnologies, and novel information technologies such as artificial intelligence and big data. The identification of important genes using genetics and transgenic methods forms the foundation of deep learning-based breeding. New technologies, including multiomics, artificial intelligence, and big data, expedite the breeding process through plant phenotypic analysis and high-throughput phenotyping platforms, facilitate the evaluation of plant materials, discovery of specific genes, and accelerated breeding (Pan, 2015; Banerjee et al., 2020). Integration of multiomics data, encompassing genomics, metabolomics, phenomics, proteomics, and transcriptomics, aids in analyzing biological changes and regulatory processes, identifying key genes and regulatory elements, and driving plant breeding (Yang et al., 2021). What’s even more exciting is that Telomere-to-Telomere (T2T) complete genome and T2T whole-genome analysis serve as representative markers for accurately identifying genetic diversity and enhancing functional genomics and genetic improvement (Deng et al., 2022). Additionally, gene editing techniques have also contributed to breeding advancements (Li et al., 2018). Intelligent breeding strategies driven by big data and artificial intelligence, enable targeted breeding, such as through comprehensive genomic and environmental prediction (iGEP) based on genomics and population-environment interactions (Yin et al., 2008). Deep learning frameworks support automatic differentiation, enabling efficient implementation of these scores with just a few lines of code. They will assist in handling extensive multidimensional big data of genotype-phenotype-environment, facilitating efficient selection and cultivation of high-quality, disease-resistant new varieties (see **Table 1**). It should be pointed out that extensive genetic experiments with correlated phenotypic and environmental data are necessary (Parmley et al., 2019; Xu et al., 2021). It is also important, especially in complex models, to indirectly examine parameters by inspecting the input-output relationships for each predicted example. Feature importance scores highlight the most influential parts of a given input for model predictions, helping to explain why such predictions are made. In DNA sequence-based models, feature importance scores highlight sequence motifs and are widely used in genomics (Alipanahi et al., 2015; Kelley et al., 2016; Kelley et al., 2018). They can also be used to explore more complex epistatic interactions (Greenside et al., 2018). Feature importance scores can be divided into two categories: perturbation-based and backpropagation-based (**Figure 2**). The former perturbs input features and observe changes in the output, but it is computationally expensive. On the other hand, the latter calculates the importance scores for all input features using a single backpropagation pass, making them computationally efficient. The simplest backpropagation-based importance scores are saliency maps (Simonyan et al., 2013) and input-masked gradients (Shrikumar et al., 2017).

**Table 1:** Deep learning algorithms and models used in intelligent breeding.

Categories (model)	Data		Application
CNN (DeepBind)	DNA sequences		DNA and Gene Characteristics (Alipanahi et al., 2015)
CNN (DeepSEA)	DNA sequences		DNA and Gene Characteristics (Zhou and Troyanskaya, 2015)
Bi-GRU and LSTM (DeepCpG)	DNA sequences		DNA and Gene Characteristics (Angermueller et al., 2017)
CNN and RNN (BiRen)	DNA sequences		DNA and Gene Characteristics (Yang et al., 2017)
CNN (Basset)	DNA sequences		DNA and Gene Characteristics (Kelley et al., 2018)
RNN	DNA sequences, DNA probes		DNA and Gene Characteristics (Zhang et al., 2021a)
ResNet and LSTM (DeepcycP)	DNA sequences		DNA and Gene Characteristics (Li et al., 2022a)
DBN	Gene sequences		DNA and Gene Characteristics (Rachmatia et al., 2017)
GCN	Genetic diagram	interaction	DNA and Gene Characteristics (Dutil et al., 2018)
CNN and LSTM (DeepNovo)	Peptide spectrum	sequences,	Protein Characteristics (Tran et al., 2017)
BiLSTM and SVM-rank (pNovo3)	Peptide spectrum	sequences,	Protein Characteristics (Yang et al., 2019)
GRU	spectrum		Protein Characteristics (Gessulat et al., 2019)
Stacked AutoEncoder and DNN	protein sequences		Protein Characteristics (Zhang and Kabuka, 2019)
RCNN	protein sequences		Protein Characteristics (Xu et al., 2021)
Evoformer and transformer (AlphaFold)	protein sequences		Protein Characteristics (Jumper et al., 2021)
GCN (Decagon)	protein-protein interaction network		Protein Characteristics (Zitnik et al., 2018)
GAN	DNA sequences		Protein Characteristics (Gupta and Zou, 2018)
Multilayer Perceptron and autoencoder	SNP		Genomics Variations (Xie et al., 2017)
GAN	RNA sequences		Genomics Variations (Ghahramani et al., 2018)
DCNN	DNA sequences		Genomics Variations (Kelley, 2020)
CNN (AMBER)	gene sequences		Genomics Variations (Zhang et al., 2021a)
RCNN	RGB image		Genomics Variations (Li et al., 2022b)
Multilayer Perceptron Classifier	DNA sequences		Genomics Variations (Chen et al., 2023)

## Applications of deep learning in crop breeding

### DNA and gene characteristics

The shape of DNA plays an important role in the specificity of transcription factor (TF)-DNA binding (Lai et al., 2019), and deep learning models can utilize various types of data for analysis (Zampieri et al., 2019). Understanding the sequence specificity of DNA and RNA binding proteins is crucial for biological system regulation models and pathogenic variant identification (Wang et al., 2020). There are currently several deep learning-based methods for predicting TF binding properties. DeepBind (Alipanahi et al., 2015), Basset (Kelley et al., 2018), and DeepSEA (Zhou and Troyanskaya, 2015) were among the earliest convolutional neural networks (CNNs) applied to genomic data analysis. DeepBind trained multiple single-task models to predict the binding affinities of transcription factors, while DeepSEA compiled a large set of chromatin maps for non-coding variants to study chromatin features, and Basset predicted DNA accessibility features. The impact of functional non-coding variants was evaluated in DeepSEA, DFIM (Greenside et al., 2018), and DeFine (Wang et al., 2018). This has always been considered a challenge to identify critical genomic regulatory regions in species with abundant repetitive elements and broad intergenic regions. To address this challenge, efficient and accurate annotation of regulatory regions in maize was achieved using methods based on natural language processing, such as k-mer grammar (Qin and Feng, 2017). These methods have played an important role in the prediction of functional non-coding variants, regulatory region annotation, and transcription factor binding site (TFBS) prediction. Machine learning models have proven to be efficient in plant biology, capable of being trained on various types of sequencing data while incorporating additional information, such as DNase I hypersensitivity data, to improve the prediction of *in vivo* transcription factor binding sites (Qin and Feng, 2017). In summary, CNNs have been widely applied in predicting molecular phenotypes from DNA sequences and have become advanced models. They have been used for classifying transcription factor binding sites (Wang et al., 2018), chromatin function (Kelley et al., 2018), DNA contact mapping (Schreiber et al., 2017), DNA methylation (Angermueller et al., 2017; Zeng and Gifford, 2017), gene expression (Zhou et al., 2018), and RNA binding protein (Pan and Shen, 2017). Additionally, CNNs have been successfully applied to tasks such as RNA specificity prediction (Kim et al., 2018) and enhancing Hi-C data resolution (Zhang et al., 2018). Not surprisingly, CNNs can model long-range dependencies in the genome and improve the accuracy of predicting molecular phenotypes from linear DNA sequences through dilated convolutions (Zeng and Gifford, 2017). Interestingly, in addition to the CNN model, several other deep learning models are also used to analyze genetic characteristics. For instance, Angermueller et al. (2017) designed the DeepCpG model based on RNN, which can predict single-cell methylation states from local DNA sequences and observed neighboring methylation states. Zhang et al. (2021a) constructed a deep learning model to predict the depth of next generation sequencing according to the DNA probe sequences. Enhancer elements are non coding fragments of DNA that play a crucial role in controlling gene expression programs. Yang et al. (2017) proposed a hybrid BiRen architecture based deep learning, which only used DNA sequences to predict enhancers. Li et al. (2022b) constructed a deep model called DeepcycP that combines the Inception ResNet structure and LSTM layer, which can predict intrinsic DNA cyclization with high fidelity. Rachmatia et al. (2017) designed a deep learning algorithm DBN, which used whole-genome single-nucleotide polymorphism (SNP) as training and testing data to construct a genome prediction model. The results showed that the DBN algorithm had a correlation of 0.579 within the range of [*−*1,1] with non additive features. Dutil et al. (2018) studied gene expression by deep learning and applied bias to the model using gene interaction maps, which has advantages in specific tasks within a low data range.

### Protein characteristics

There is a close relationship between the function and structure of proteins. The function of a protein is determined by its tertiary structure, which can be revealed through comprehensive analysis of various protein characteristics. To extract important amino acid features from primary peptide sequences, DeepNovo (Tran et al., 2017) was developed using the CNN method. pNovo3 (Yang et al., 2019) utilizes a learning-to-rank framework to differentiate similar peptide candidates for each spectrum. It employs three metrics to measure the similarity between experimental and theoretical spectra, with the theoretical spectra precisely predicted through deep learning using the pDeep algorithm. In mass spectrometry-based proteomics, identification and quantification of peptides and proteins heavily rely on database searching and spectrum matching. The lack of accurate models for predicting fragment ion intensities limits the practicality of these methods. By expanding the ProteomeTools synthetic peptide library and training the deep neural network Prosit, the prediction accuracy of chromatographic retention time and fragment ion intensities has been significantly improved (Gessulat et al., 2019). Gupta and Zou (2018) used GAN to generate DNA sequences for proteins with variable coding lengths, which have ideal biophysical properties. Protein-protein interactions are crucial for understanding biological processes and disease mechanisms. Researchers have explored various methods to predict protein-protein interactions, including sequence-based prediction techniques (Hashemifar et al., 2018) and deep learning models (Mirabello and Wallner, 2018). One approach involves unsupervised derivation of novel protein features from the “proteinprotein” interaction network, followed by using these features to predict protein functions in different tissues (Zitnik and Leskovec, 2017). Zitnik et al. (2018) proposed the graph convolutional neural network model Decagon, and used this model to construct multimodal graphs of protein protein interactions, drug protein target interactions, and multi drug side effects.Some of these methods also incorporate physicochemical features of proteins and topological features of protein-protein interaction (PPI) networks to enhance predictive performance through multimodal supervised deep representation learning (Zhang and Kabuka, 2019). A novel residue representation method called Res2vec has been designed to represent protein sequences, combining effective feature embedding and powerful deep learning techniques, providing a universal computational pipeline for inferring “protein-protein” interactions (Longwell and Shimko, 2022). The confidence score of a protein sequence pair can be regarded as a measure of PPI. Therefore, a deep learning framework (Xu et al., 2021), namely the ordinal regression and recursive convolutional neural network approach, has been introduced to predict PPI from the perspective of confidence. Analysis of co-variation in homologous sequences aids in predicting protein structures. AlphaFold is an algorithm that predicts protein structures using deep learning methods, training neural networks to predict distances between residues and generate protein structures (Jumper et al., 2021). AlphaFold2 is an improved version of AlphaFold, greatly enhancing the accuracy of protein structure prediction by introducing new neural network architectures and training procedures (Tunyasuvunakool et al., 2021).

### Genomics variations

Despite the presence of numerous genetic variations in natural populations, it is possible to train deep learning models on a small subset of these variations to predict the effects across the entire spectrum of mutations (Killamsetty et al., 2021). For instance, models trained on certain genes can be used to predict the outcomes of other genes. These models encompass various types of mutations, including common alleles as well as rare and low-frequency variants, regardless of their impact on gene function. Xie et al. (2017) constructed a deep automatic encoder model to evaluate the impact of genetic variation on gene expression changes. Li et al. (2022b) developed an image-based wheat spike counter using the Faster R-CNN algorithm, revealing significant differences between genotypes. ExPecto is a deep learning framework that accurately predicts the transcriptional effects of mutations in DNA sequences, including rare or unobserved mutations (Zhou et al., 2018). This framework enables initial predictions for exploring the evolutionary constraints on gene expression and the effects of mutational diseases. Furthermore, models trained in one species can be directly applied to closely related species (Kelley, 2020), due to the conservation of molecular processes in closely related species. Chen et al. (2023) proposed an unsupervised clustering method and developed a deep learning model accordingly to predict gene mutations. Ghahramani et al. (2018) used GAN to simulate gene expression and predict perturbations in single cells, thereby identifying biological state determining genes and ultimately inferring gene regulatory relationships. A biologically-informed automated modeling framework, known as AMBER (Zhang et al., 2021b), has been proposed. It is a fully automated framework that efficiently designs and applies CNNs to genomic sequences. AMBER utilizes state-of-the-art neural architecture search to design optimal models for specified biological questions. Applying AMBER to modeling tasks of genomic regulatory features has demonstrated significantly more accurate predictions compared to non-neural architecture search baseline models, matching or even surpassing expert-designed models. In summary, deep learning models have the potential to greatly advance our understanding of genomic variations in relation to the ultimate phenotypes.

## The impact on crop quality and yield of deep learning-empowered crop breeding

Climate change is seriously hindering the development of agricultural productivity globally, with significant impacts on crop yield and quality (Praveen and Sharma, 2019). Analyzing and identifying crop images using deep learning models can aid in rapidly identifying superior plants with target traits, thereby accelerating the process of crop breeding and selecting high-yielding, disease-resistant, and other desirable varieties. Deep learning models can also recognize crop performance under adverse environmental conditions such as drought and salinity stress, helping to cultivate more resilient crop varieties (Sun et al., 2019). Specifically, the identification and classification algorithm for corn leaf blight achieved high accuracy using the CNN algorithm, which is of significant importance for rapid detection of crop diseases and improving crop yields (Abdullahi et al., 2017). The solutions for crop disease identification and diagnosis were provided using different deep learning methods (Ferentinos, 2018). In terms of abiotic stress, the extraction of time-series chlorophyll fluorescence features using the SAE neural network algorithm provided an effective means for identifying chlorophyll fluorescence fingerprints in *Arabidopsis thaliana*, offering new insights for improving crop drought resistance (Sun et al., 2019). By using a large number of soybean leaf images for deep learning classification, the identification and classification of soybean symptoms under non-biological stress was achieved, enabling rapid detection of non-biological stress in soybeans (Ghosal et al., 2018). The good correlation between the classification of corn freeze damage based on spectral features of multiple genotypes and the discrimination results based on chemical values was demonstrated using a CNN model (Yang et al., 2019). Employing various deep learning methods for diagnosing pumpkin leaf diseases helps farmers detect crop damage early (Nirmala and Gomathy, 2019). Using an integrated classifier based on a deep convolutional neural network for identifying citrus pests has effectively enhanced the quality and yield of citrus fruits (Khanramaki et al., 2021). Developing a model to estimate the number of leaves and plant age for watermelon plants, and classifying them under normal and low-temperature stress, facilitates growth monitoring and improves water and sugar content in watermelons (Nabwire et al., 2022). Training deep learning models to classify coffee leaf images and determine if they are infected with leaf rust disease can aid in early detection of diseases and enables timely measures to protect coffee crop yields and quality (Shao et al., 2022). Furthermore, deep learning can analyze the correlation between phenotypic genomic, facilitating precise selection and optimization of genomic combinations as well as gene editing techniques to improve crop yields and quality. For instance, deep learning-based genomic selection models (GS) have shown outstanding performance in predicting wheat terminal quality traits, advancing the deployment of superior genotypes into broader grain yield trials (Sandhu et al., 2021). Therefore, deep learning can analyze massive amounts of data, build intelligent breeding decisions, and rapidly create superior inbred lines, effectively shortening breeding cycles, improving breeding efficiency, reducing costs, and enhancing crop yields and quality.

## Challenges and prospects

In the past 20 years, machine learning has achieved significant success in the field of agriculture. In recent years, deep learning, as a branch of machine learning, has represented the most advanced technology in smart agriculture (Kamilaris and Prenafeta-Boldú, 2018). As an integral part of agriculture, deep learning has been widely applied to various plant phenomics, such as image classification (Ramcharan et al., 2017), object detection (Ghosal et al., 2019), and semantic segmentation (Aich and Stavness, 2017). Consequently, it has tremendous potential in predicting plant growth, estimating yield, detecting maturity, and perceiving biotic/abiotic stresses. However, deep learning algorithms require a large amount of labeled data (Cordts et al., 2016), and data acquisition comes at a high cost, especially when identifying numerous categories (Tong et al., 2022) or subtle differences between categories (Taghavi Namin et al., 2018). Furthermore, collecting phenotype data faces additional obstacles of severe occlusion and various lighting conditions (Scharr et al., 2016), which increase the time required for obtaining the necessary annotations. Genotypic, phenotypic, and environmental big data form the core of intelligent breeding design (Talbot et al., 2017). Nevertheless, there is a significant shortage of accumulated phenotype data, and the problems with traditional manual detection are increasingly prominent, necessitating a balanced consideration of accuracy, throughput, and cost (Liu and Wang, 2021). It is anticipated that breakthroughs and innovations in next-generation sensors and robotics will serve as underlying driving technologies to accelerate the acquisition of crop phenotype big data (Sony et al., 2019). By utilizing bio-sensors and agricultural robots, continuous detection of multiple traits is achieved, leading to improved detection accuracy, but the development of sensors and robots also faces certain challenges. Firstly, the working mechanisms and conditions may vary significantly. Even within the same species and variety, robot components may require adjustments or replacements (De Preter et al., 2018), reducing the universality of robots. Most studies only report simulations, experiments, preliminary results, and specifications related to hardware/software design. In contrast, only a few studies discuss commercial solutions (Bagagiolo et al., 2022). Additionally, the efficiency of the sensors and robots used is not high. If local labor is inexpensive, there is an unacceptable risk associated with using sensors and robots. Currently, a better solution may be to enable collaboration between workers and robots (Bragança et al., 2019).

The availability of massive big data enables informed decision-making, however the adaptability of deep learning models across different crops and environmental conditions may be limited (Khaki and Wang, 2019). Due to the differences in crop genetics and environmental factors, model transfer from one crop to another may require additional adjustments and optimization (Ghazi et al., 2017). This poses challenges for the application of deep learning in intelligent analysis and interpretation. Big data includes plant phenotypes, genetic genotypes, environmental parameters, diseases, pest conditions, and more. What’s frustrating is that, the acquisition and processing of plant data lag behind research needs, limiting the development of intelligent breeding and functional plant genomics. Furthermore, they are not organically integrated, which hinders informed decision-making. In the future, researchers should collectively strive to establish a large-scale database, and interdisciplinary collaboration and data sharing can unlock greater potential for deep learning in breeding applications, benefiting more people from big data (Kim, 2019). In addition, transfer learning (Pan and Yang, 2009) and few-shot learning (Snell et al., 2017) will be effective approaches to alleviate the deep learning models’ dependency on massive datasets. Transfer learning aims to transfer knowledge accumulated from a source task with ample labeled data to a new or similar target task, particularly when training data is limited. Notably, when the source and target domains exhibit strong similarity, transfer learning can provide a more economical and expedited solution to address the constraints of scarce training data (Sun et al., 2018). The most distinct characteristic of few-shot learning is its capacity for “learning to learn”, achieved by emulating human-level concept learning, meaning that acquiring new concepts requires only a small number of labeled examples (Chen et al., 2019). Approaches like data augmentation (Shorten and Khoshgoftaar, 2019), image segmentation (Minaee et al., 2021), and attention mechanisms (Niu et al., 2021) can be used to solve the problem of severe occlusion in collected phenotype data, and improve the performance of deep learning models when facing such problems. Deep reinforcement learning is the process of making intelligent decisions through reinforcement learning on the basis of deep learning (Shaikh et al., 2022). By using deep reinforcement learning to plan the robot’s path and make decisions on its actions during its journey, the robot can efficiently assist farmers in crop data collection, crop picking, transportation, watering, and fertilization operations. No doubt, addressing key issues related to accurate collection, intelligent analysis of crop deep phenotype, and intelligent decision-making for precision agriculture on this basis will be of significant importance to the research of intelligent breeding.

## Data availability statement

The original contributions presented in the study are included in the article/supplementary material. Further inquiries can be directed to the corresponding author.

## Ethics statement

Written informed consent was obtained from the individual(s) for the publication of any identifiable images or data included in this article.

## Author contributions

XW: Formal Analysis, Software, Visualization, Writing – original draft. HZ: Formal Analysis, Software, Visualization, Writing – original draft. LL: Visualization, Writing – original draft. YH: Visualization, Writing – original draft. HL: Conceptualization, Methodology, Supervision, Visualization, Writing – original draft. YQ: Conceptualization, Funding acquisition, Project administration, Resources, Supervision, Writing – review & editing.
